# Risk factors for postoperative cervical haematoma in patients undergoing thyroidectomy: a retrospective, multicenter, international analysis (REDHOT study)

**DOI:** 10.3389/fsurg.2023.1278696

**Published:** 2023-10-02

**Authors:** Gian Luigi Canu, Fabio Medas, Federico Cappellacci, Leonardo Rossi, Benard Gjeloshi, Luca Sessa, Francesco Pennestrì, Reza Djafarrian, Maria Mavromati, George Kotsovolis, Ioannis Pliakos, Giacomo Di Filippo, Giovanni Lazzari, Carla Vaccaro, Martina Izzo, Francesco Boi, Paolo Brazzarola, Francesco Feroci, Marco Stefano Demarchi, Theodossios Papavramidis, Gabriele Materazzi, Marco Raffaelli, Pietro Giorgio Calò, REDHOT Study Collaborative Group, Giacomo Anedda

**Affiliations:** ^1^ Department of Surgical Sciences, University of Cagliari, Monserrato, Italy; ^2^ Endocrine Surgery Unit, University Hospital of Pisa, Pisa, Italy; ^3^ UOC di Chirurgia Endocrina e Metabolica, Fondazione Policlinico Universitario Agostino Gemelli IRCCS, Rome, Italy; ^4^ Centro di Ricerca in Chirurgia Delle Ghiandole Endocrine e Dell’Obesità, Università Cattolica del Sacro Cuore, Rome, Italy; ^5^ Department of Thoracic and Endocrine Surgery and Faculty of Medicine, University Hospitals of Geneva, Geneva, Switzerland; ^6^ Service of Endocrinology, Diabetes, Nutrition and Therapeutic Patient Education, WHO Collaborating Center, Geneva University Hospital, Geneva University, Geneva, Switzerland; ^7^ First Propedeutic Department of Surgery, Aristotle University of Thessaloniki, AHEPA University Hospital, Thessaloniki, Greece; ^8^ Unit of Minimally Invasive Surgery, Euromedica Kyanous Stavros, Thessaloniki, Greece; ^9^ Endocrine Surgery Unit, Department of Surgery and Oncology, University and Hospital Trust of Verona, Verona, Italy; ^10^ SOC Chirurgia Generale, Ospedale SS Cosma e Damiano, Pescia, Italy; ^11^ Department of Medical Sciences, University of Cagliari, Monserrato, Italy; ^12^ Department of General and Oncologic Surgery, Santo Stefano Hospital, Prato, Italy

**Keywords:** thyroidectomy, thyroid surgery, cervical haematoma, bleeding, risk factors, complications

## Abstract

**Background:**

Postoperative cervical haematoma represents an infrequent but potentially life-threatening complication of thyroidectomy. Since this complication is uncommon, the assessment of risk factors associated with its development is challenging. The main aim of this study was to identify the risk factors for its occurrence.

**Methods:**

Patients undergoing thyroidectomy in seven high-volume thyroid surgery centers in Europe, between January 2020 and December 2022, were retrospectively analysed. Based on the onset of cervical haematoma, two groups were identified: Cervical Haematoma (CH) Group and No Cervical Haematoma (NoCH) Group. Univariate analysis was performed to compare these two groups. Moreover, employing multivariate analysis, all potential independent risk factors for the development of this complication were assessed.

**Results:**

Eight thousand eight hundred and thirty-nine patients were enrolled: 8,561 were included in NoCH Group and 278 in CH Group. Surgical revision of haemostasis was performed in 70 (25.18%) patients. The overall incidence of postoperative cervical haematoma was 3.15% (0.79% for cervical haematomas requiring surgical revision of haemostasis, and 2.35% for those managed conservatively). The timing of onset of cervical haematomas requiring surgical revision of haemostasis was within six hours after the end of the operation in 52 (74.28%) patients. Readmission was necessary in 3 (1.08%) cases. At multivariate analysis, male sex (*P* < 0.001), older age (*P* < 0.001), higher BMI (*P* = 0.021), unilateral lateral neck dissection (*P* < 0.001), drain placement (*P* = 0.007), and shorter operative times (*P* < 0.001) were found to be independent risk factors for cervical haematoma.

**Conclusions:**

Based on our findings, we believe that patients with the identified risk factors should be closely monitored in the postoperative period, particularly during the first six hours after the operation, and excluded from outpatient surgery.

## Introduction

In endocrine surgery, thyroidectomy is the most frequently performed surgical procedure (1–3).

Morbidity related to thyroidectomy is mainly represented by hypoparathyroidism, recurrent laryngeal nerve injury, and cervical haematoma. These complications can occur at a considerable rate even if thyroid surgery is performed by highly experienced surgeons (4–7).

Obtaining accurate haemostasis during thyroidectomy is crucial to prevent the occurrence of postoperative bleeding and, allowing adequate vision of the anatomical structures, is also important to avert the onset of the other complications (7).

As regards postoperative cervical haematoma, it represents an infrequent but potentially life-threatening complication, occurring in up to 6.5% of patients (7–27).

This complication can lead to acute airway obstruction, through direct compression or venous congestion resulting in significant airway oedema, which can be followed by severe neurological sequelae or even death, if its occurrence is not promptly and appropriately managed. Cervical haematoma with associated airway compromise requires an immediate surgical revision of haemostasis. In the event of acute respiratory distress, the wound should be opened immediately at the patient's bedside. However, it is important to point out that in several cases cervical haematomas can be managed non-surgically (7–27).

Regarding the timing of onset, this complication mostly occurs within the first 6 h after surgery and is quite infrequent after 24 h (8, 10, 11, 16, 17, 20, 21, 23).

The risk of postoperative cervical haematoma, with its possible consequences, is the most significant obstacle to the expansion of outpatient thyroidectomy, which is becoming increasingly popular in recent years (10, 11, 26). A better understanding of the risk factors for the occurrence of this complication may help to select patients suitable for outpatient thyroid surgery. However, since cervical haematoma is uncommon, the assessment of risk factors associated with its development is challenging (8–27).

The main aim of this study was to identify the risk factors for the onset of postoperative cervical haematoma in patients undergoing thyroidectomy.

## Materials and methods

### Study design and population

This is a multicenter, retrospective, international study on patients undergoing thyroidectomy between January 2020 and December 2022.

Data were collected from seven high-volume thyroid surgery centers in Europe:

- General Surgery Unit, Cagliari University Hospital, Monserrato, Italy;
- Endocrine Surgery Unit, Pisa University Hospital, Pisa, Italy;
- Endocrine and Metabolic Surgery Unit, “Fondazione Policlinico Universitario A. Gemelli

IRCCS”, Rome, Italy;

- Minimally Invasive Surgery Unit, Euromedica Kyanous Stavros, Thessaloniki, Greece;
- Thoracic and Endocrine Surgery Division, Geneva University Hospitals, Geneva, Switzerland;
- Endocrine Surgery Unit, Verona University Hospital, Verona, Italy;
- General Surgery Unit, Prato Hospital “Santo Stefano”, Prato, Italy.

All participating centres used the same dedicated Microsoft Office Excel database (Microsoft Corporation, Redmond, WA, USA) for data collection.

Patients submitted to total thyroidectomy, hemithyroidectomy, and completion thyroidectomy, with or without neck dissection or simultaneous parathyroidectomy, were included in this analysis.

Exclusion criteria were: age <18 years, patients with coagulation disorders (involving platelets and/or coagulation factors), remote surgical approaches (transoral endoscopic thyroidectomy vestibular approach and robot-assisted transaxillary thyroidectomy), and incomplete data.

Based on the occurrence of cervical haematoma, two groups were identified: Cervical Haematoma (CH) Group and No Cervical Haematoma (NoCH) Group.

Demographic and preoperative data, details about the operation, postoperative stay, histological diagnosis, and the other early complications of thyroid surgery were assessed.

### Endpoints

The primary endpoint was to identify the risk factors for the occurrence of postoperative cervical haematoma.

As secondary endpoints, the incidence of cervical haematoma, the rate of readmission for this complication, the timing of onset of haematomas requiring surgical revision of haemostasis, and the occurrence of the other early complications of thyroidectomy (recurrent laryngeal nerve injury, postoperative hypoparathyroidism, and wound infection) were evaluated.

### Preoperative features and management of antithrombotic drugs

Hyperthyroidism status was defined in the case of low serum thyroid-stimulating hormone (TSH) (<0.4 mIU/L) and use of thyrostatic drugs.

Retrosternal goiter was defined as a thyroid in which any part of the gland extended below the thoracic inlet with the patient in the surgical position.

Antiplatelet drugs, depending on the patient's thrombotic risk, the preference of the operating surgeon and the practice of the center, were continued during the perioperative period or discontinued 7–10 days before surgery. In patients taking dual antiplatelet therapy, acetylsalicylic acid was continued during the perioperative period while P2Y_12_ receptor inhibitors (such as clopidogrel, ticlopidine, ticagrelor or prasugrel) were discontinued 5–7 days before surgery. These patients were considered among those who did not discontinue the antiplatelet drug.

Vitamin K antagonists (VKAs) were discontinued approximately 5 days before surgery and replaced with therapeutic-dose low-molecular-weight heparin (LMWH) during the perioperative period.

Direct oral anticoagulants (DOACs) were discontinued 48–72 h before surgery and reintroduced approximately 48 h after the operation, without using bridging anticoagulation.

### Surgical procedure

Thyroidectomies were performed by conventional open or minimally invasive video-assisted approach.

Parathyroid glands and recurrent laryngeal nerves were systematically searched and identified.

Energy-based devices (working by means of ultrasonic, advanced bipolar or hybrid energy), intermittent or continuous intraoperative nerve monitoring (IONM), and topical haemostatic agents were used according to the preference of the operating surgeon or depending on their availability.

Drain placement was at the surgeon's discretion.

The duration of surgery was estimated, in minutes, from skin incision to skin closure.

### Assessment of complications

Postoperative cervical haematomas were distinguished according to whether or not surgical revision of haemostasis was necessary. The timing of onset of cervical haematomas requiring surgical revision of haemostasis was assessed, in hours, from the end of surgery to the diagnosis of the complication. In this regard, three periods were identified: within six hours after the end of the operation, between 7 and 24 h and after 24 h.

Serum calcium and iPTH levels were assessed pre and postoperatively. Postoperative hypoparathyroidism was defined as iPTH < 10 pg/ml after surgery (normal range = 10–65 pg/ml).

Preoperative fibrolaryngoscopy was always performed to assess vocal fold mobility. Recurrent laryngeal nerve injury was diagnosed through postoperative fibrolaryngoscopy. After the operation, fibrolaryngoscopy was performed in all patients with suspected recurrent laryngeal nerve injury for loss of signal at IONM or hoarseness.

### Statistical analysis

Statistical analyses were performed with MedCalc® version 22.009.

Univariate analysis was performed to compare the two groups. Fisher exact test or Chi-squared test were utilized, as appropriate, for categorical variables. The presence of a normal distribution of continuous variables was assessed using the D'Agostino-Pearson test. Based on the results of the latter test, Mann-Whitney U test was employed for continuous variables, which were expressed as median and interquartile range (IQR).

Employing multivariate analysis (using the “Enter” method), all potential independent risk factors for postoperative cervical haematoma were assessed.

*P* values were considered statistically significant if <0.05.

## Results

Based on the inclusion criteria, 8,839 patients were enrolled: 8,561 were included in NoCH Group and 278 in CH Group.

Surgical revision of haemostasis was performed in 70 (25.18%) patients, while in the other 208 (74.82%) cases cervical haematoma was managed conservatively.

The overall incidence of this complication was 3.15%. In particular, for cervical haematomas requiring surgical revision of haemostasis the incidence was 0.79%, while for those managed conservatively 2.35%.

**Figure 1:**
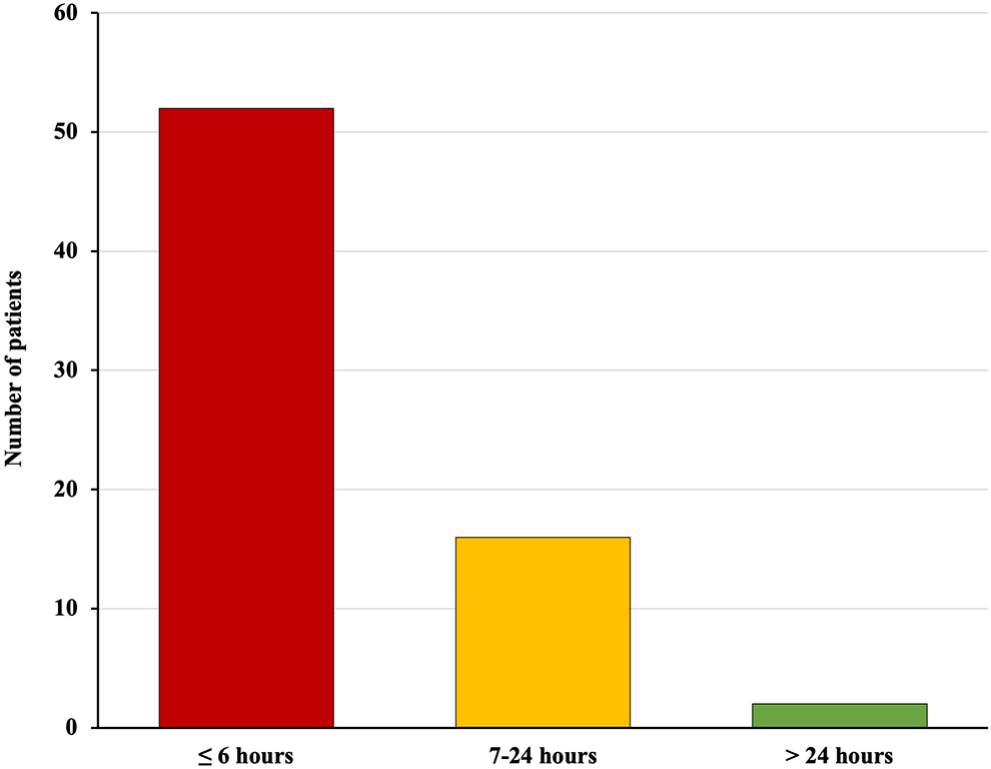
Timing of onset of postoperative cervical haematomas requiring surgical revision of haemostasis (from the end of surgery to the diagnosis of the complication): within six hours in 52 (74.28%) patients, between 7 and 24 h in 16 (22.86%), and after 24 h in 2 (2.86%).

The timing of onset of cervical haematomas requiring surgical revision of haemostasis was: within six hours after the end of the operation in 52 (74.28%) patients, between 7 and 24 hours in 16 (22.86%), and after 24 h in 2 (2.86%) (Figure 1).

Readmission for this complication was necessary in 3 (1.08%) cases. In one of these patients it was necessary to perform a surgical revision of the haemostasis, while in the other 2 the management was conservative.

### Demographic data, preoperative features and histopathological diagnosis

Statistically significant differences between the two groups were found in terms of sex, age, BMI, and high blood pressure. The other variables (hyperthyroidism, use of antithrombotic drugs, retrosternal goiter, and histological diagnosis) were comparable.

Detailed results are shown in Table 1.

**Table 1:** Demographic data, preoperative features, and histological diagnosis.

** **	Total (*n* = 8,839)	NoCH group (*n* = 8,561)	CH group (*n* = 278)	*P* value
Sex				
Male	2,300 (26.02%)	2,191 (25.59%)	109 (39.21%)	**<0** **.** **001**
Female	6,539 (73.98%)	6,370 (74.41%)	169 (60.79%)	
Age (years, median and IQR)	52 (41–62)	52 (41–62)	57 (46–66)	**<0** **.** **001**
BMI (kg/m^2^, median and IQR)	25 (22.66–29)	25 (22.60–28.82)	26 (24–30)	**<0** **.** **001**
High blood pressure	2,293 (25.94%)	2,183 (25.50%)	110 (39.57%)	**<0** **.** **001**
Hyperthyroidism	1,583 (17.91%)	1,531 (17.88%)	52 (18.71%)	0.725
Use of antiplatelets				
No	8,306 (93.97%)	8,054 (94.08%)	252 (90.64%)	0.060
Yes, discontinued	320 (3.62%)	304 (3.55%)	16 (5.76%)	
Yes, not discontinued	213 (2.41%)	203 (2.37%)	10 (3.60%)	
Use of VKAs				
No	8,821 (99.80%)	8,543 (99.79%)	278 (100%)	1.000
Yes	18 (0.20%)	18 (0.21%)	0	
Use of DOACs				
No	8,684 (98.25%)	8,415 (98.29%)	269 (96.76%)	0.062
Si	155 (1.75%)	146 (1.71%)	9 (3.24%)	
Retrosternal goiter	442 (5.0%)	432 (5.05%)	10 (3.60%)	0.275
Histological diagnosis				
Graves’ disease	788 (8.96%)	761 (8.89%)	27 (9.71%)	0.636
Hashimoto's thyroiditis	2,484 (28.10%)	2,418 (28.24%)	66 (23.74%)	0.100
Malignancy	4,097 (46.35%)	3,980 (46.49%)	117 (42.09%)	0.147

IQR, interquartile range; BMI, body mass index; VKAs, vitamin K antagonists; DOACs, direct oral anticoagulants.

Statistically significant values are indicated in bold.

### Information about the surgical procedure and postoperative stay

Statistically significant differences between the two groups were found in terms of lateral neck dissection, volume of surgeons, use of IONM, haemostasis technique, drain placement, operative time, and postoperative stay. The other variables (type of thyroidectomy, central neck dissection, parathyroidectomy, MIVAT, and topical haemostatic agents) were comparable.

Detailed results are reported in Table 2.

**Table 2:** Details about the operation and postoperative stay.

** **	Total (*n *= 8,839)	NoCH group (*n* = 8,561)	CH group (*n* = 278)	*P* value
Type of thyroidectomy				
TT	6,937 (78.48%)	6,708 (78.36%)	229 (82.37%)	0.206
HT	1,666 (18.85%)	1,625 (18.98%)	41 (14.75%)	
CT	236 (2.67%)	228 (2.66%)	8 (2.88%)	
Central neck dissection				
No	7,845 (88.75%)	7,599 (88.76%)	246 (88.49%)	0.578
Unilateral	158 (1.79%)	155 (1.81%)	3 (1.08%)	
Bilateral	836 (9.46%)	807 (9.43%)	29 (10.43%)	
Lateral neck dissection				
No	8,444 (95.53%)	8,188 (95.64%)	256 (92.09%)	**0**.**016**
Unilateral	348 (3.94%)	328 (3.83%)	20 (7.19%)	
Bilateral	47 (0.53%)	45 (0.53%)	2 (0.72%)	
Parathyroidectomy	160 (1.81%)	156 (1.82%)	4 (1.44%)	0.637
MIVAT	72 (0.81%)	70 (0.82%)	2 (0.72%)	1.000
Volume of surgeons^a^				
>50	7,902 (89.40%)	7,638 (89.22%)	264 (94.96%)	**0**.**004**
25–50	174 (1.97%)	174 (2.03%)	0	
<25	763 (8.63%)	749 (8.75%)	14 (5.04%)	
Use of IONM				
No	5,650 (63.92%)	5,406 (63.15%)	244 (87.77%)	**<0** **.** **001**
Intermittent IONM	2,850 (32.24%)	2,817 (32.90%)	33 (11.87%)	
Continuous IONM	339 (3.84%)	338 (3.95%)	1 (0.36%)	
Haemostasis technique				
Advanced bipolar EBD	4,773 (54.0%)	4,620 (53.96%)	153 (55.04%)	**<0** **.** **001**
Ultrasonic EBD	1,502 (17.0%)	1,474 (17.22%)	28 (10.07%)	
Hybrid EBD	262 (2.96%)	261 (3.05%)	1 (0.36%)	
Conventional	2,302 (26.04%)	2,206 (25.77%)	96 (34.53%)	
Use of haemostatic agents	2,645 (29.92%)	2,571 (30.03%)	74 (26.62%)	0.221
Drain placement	6,856 (77.57%)	6,606 (77.16%)	250 (89.93%)	**<0** **.** **001**
Operative time (minutes, median and IQR)	60 (45–90)	60 (45–90)	55 (45–75)	**<0** **.** **001**
Postoperative stay (days, median and IQR)	1 (1–2)	1 (1–2)	2 (1–3)	**<0** **.** **001**

TT, total thyroidectomy; HT, hemithyroidectomy; CT, completion thyroidectomy; MIVAT, minimally invasive video-assisted thyroidectomy; IONM, intraoperative nerve monitoring; EBD, energy-based device; IQR, interquartile range.

^a^ Thyroidectomies per year.

Statistically significant values are indicated in bold.

### Other early complications

The rate of postoperative hypoparathyroidism (12.28% in NoCH Group vs. 6.83% in CH Group, *P* = 0.006) was significantly inferior in CH Group than in NoCH Group, while unilateral RLN lesions (2.94% in NoCH Group vs. 5.04% in CH Group, *P* = 0.045) were significantly greater in CH Group than in NoCH Group.

No statistically significant difference was found in terms of bilateral RLN injury and wound infection.

Detailed results are shown in Table 3.

**Table 3:** Other early complications.

** **	Total (*n* = 8,839)	NoCH group (*n* = 8,561)	CH group (*n* = 278)	*P* value
Postoperative hypoparathyroidism	1,070 (12.11%)	1,051 (12.28%)	19 (6.83%)	**0**.**006**
RLN injury				
Unilateral	266 (3.01%)	252 (2.94%)	14 (5.04%)	**0**.**045**
Bilateral	14 (0.16%)	12 (0.14%)	2 (0.72%)	0.070
Wound infection	22 (0.25%)	20 (0.23%)	2 (0.72%)	0.151

RLN, recurrent laryngeal nerve.

Statistically significant values are indicated in bold.

### Multivariate analysis

In this analysis, male sex (*P* < 0.001), older age (*P* < 0.001), higher BMI (*P* = 0.021), unilateral lateral neck dissection (*P* < 0.001), drain placement (*P* = 0.007), and shorter operative times (*P* < 0.001) were found to be independent risk factors for the onset of postoperative cervical haematoma.

Differently, the use of EBD working by means of hybrid energy (*P* = 0.025) was found to be protective for the development of this complication.

Detailed results are reported in Table 4.

**Table 4:** Multivariate analysis of risk factors for postoperative cervical haematoma.

** **	Regression coefficient	Odds ratio	95% CI	*P* value
Male sex	0.609	1.839	1.415–2.390	**<0** **.** **001**
Age	0.018	1.018	1.007–1.028	**<0** **.** **001**
BMI	0.027	1.028	1.004–1.052	**0**.**021**
High blood pressure	0.270	1.310	0.980–1.750	0.068
Hyperthyroidism	−0.014	0.986	0.636–1.529	0.949
Use of antiplatelets				
No	1.000	1.000	Reference	
Yes, discontinued	0.112	1.119	0.650–1.926	0.686
Yes, not discontinued	−0.051	0.951	0.486–1.861	0.883
Use of VKAs	−20.456	<0.001		0.999
Use of DOACs	0.205	1.228	0.596–2.531	0.578
Retrosternal goiter	−0.312	0.732	0.377–1.421	0.357
Type of thyroidectomy				
TT	1.000	1.000	Reference	
HT	−0.277	0.758	0.522–1.100	0.145
CT	0.102	1.108	0.529–2.319	0.786
Central neck dissection				
No	1.000	1.000	Reference	
Unilateral	0.246	1.278	0.389–4.205	0.686
Bilateral	0.036	1.037	0.606–1.773	0.895
Lateral neck dissection				
No	1.000	1.000	Reference	
Unilateral	1.239	3.451	1.860–6.403	**<0** **.** **001**
Bilateral	1.211	3.358	0.715–15.766	0.125
Parathyroidectomy	−0.004	0.996	0.361–2.749	0.994
MIVAT	0.997	2.710	0.605–12.128	0.192
Volume of surgeons^a^				
>50	1.000	1.000	Reference	
25–50	−20.277	<0.001		0.998
<25	0.237	1.267	0.646–2.484	0.491
Haemostasis technique				
Advanced bipolar EBD	1.000	1.000	Reference	
Ultrasonic EBD	0.021	1.021	0.643–1.621	0.929
Hybrid EBD	−2.257	0.105	0.015–0.754	**0**.**025**
Conventional	0.235	1.264	0.953–1.678	0.104
Use of haemostatic agents	0.131	1.140	0.835–1.554	0.410
No drain placement	−0.719	0.487	0.289–0.823	**0**.**007**
Operative time	−0.011	0.990	0.985–0.994	**<0** **.** **001**
Graves’ disease	0.322	1.380	0.774–2.459	0.275
Hashimoto's thyroiditis	−0.064	0.938	0.698–1.262	0.673
Malignancy	−0.234	0.791	0.601–1.042	0.096

BMI, body mass index; VKAs, vitamin K antagonists; DOACs, direct oral anticoagulants; TT, total thyroidectomy; HT, hemithyroidectomy; CT, completion thyroidectomy; MIVAT, minimally invasive video-assisted thyroidectomy; EBD, energy-based device.

^a^ Thyroidectomies per year.

Statistically significant values are indicated in bold.

## Discussion

To date, several risk factors for the development of postoperative cervical haematoma have been reported in the literature: older age, male gender, higher body mass index, high blood pressure, postoperative coughing and vomiting, active smoking, use of antithrombotic drugs (antiplatelet agents and/or anticoagulants), previous thyroid surgery, extent of surgery (bilateral thyroidectomy, neck dissection), low surgeon experience, low-volume hospitals, use of topical haemostatic agents, drain placement, retrosternal goiter, large thyroid gland, thyroid malignancy, Graves’ disease, and Hashimoto's thyroiditis (8–27).

The main aim of this study was to identify the risk factors for the occurrence of postoperative cervical haematoma. Moreover, as secondary endpoints, the incidence of cervical haematoma, the rate of readmission for this complication, the timing of onset of haematomas requiring surgical revision of haemostasis, and the occurrence of the other early complications of thyroidectomy (recurrent laryngeal nerve injury, postoperative hypoparathyroidism and wound infection) were assessed.

Our overall incidence of postoperative cervical haematoma was 3.15%, while rates of cervical haematomas requiring surgical revision of haemostasis and those managed conservatively were 0.79% and 2.35%, respectively. In most cases (74.82%), it was possible to manage this complication conservatively. The timing of onset of cervical haematomas requiring surgical revision of haemostasis was within six hours after the end of the operation in 74.28% of patients, and the rate of readmission was 1.08%.

Our findings regarding the incidence rates of this complication and the timing of onset of cervical haematomas requiring surgical revision of haemostasis are in accordance with those reported by other authors. With regard to the percentage of cervical haematomas managed conservatively, it is not possible to make an adequate comparison with other studies, as the vast majority of these only consider cervical haematomas requiring surgical revision of haemostasis. Our readmission rate is also not adequately comparable, as data in the literature are very lacking (8–27).

Regarding our primary endpoint, our study identified male sex, older age, higher BMI, unilateral lateral neck dissection, drain placement, and shorter operative times as independent risk factors for postoperative cervical haematoma. Differently, concerning haemostasis techniques, the use of energy-based devices working by means of hybrid energy was found to be protective for the development of this complication.

Drain placement, in our opinion and according to other authors (21), rather than a factor causing the cervical haematoma, probably reflects the surgeon's increased concern about the development of this complication due to bleeding during surgery.

To our knowledge, our findings regarding operative times and the use of energy-based devices working by means of hybrid energy have not been described in other studies (8–27).

With regard to our result relating to operative times, we hypothesised that it might be due to the fact that adequate time was probably not devoted to the final control of haemostasis.

About our result regarding the use of energy-based devices working by means of hybrid energy, in our opinion, it is probably due to the fact that this type of devices integrates two different forms of energy, thus achieving better haemostasis (7, 28, 29).

As regards the occurrence of the other early complications of thyroidectomy, which was one of our secondary endpoints, no statistically significant difference was found in terms of bilateral recurrent laryngeal nerve injury and wound infection. Differently, in patients who developed cervical haematoma, the rate of postoperative hypoparathyroidism was significantly inferior (12.28% vs. 6.83%), while unilateral recurrent laryngeal nerve lesions (2.94% vs. 5.04%) were significantly greater.

In the literature, in the study by Burkey et al. (8), the overall occurrence of additional complications was significantly greater in patients who developed cervical haematoma. However, the frequency of individual complications was comparable. In the study by de Carvalho et al. (23), significantly greater rates of transient hypocalcemia, permanent hypoparathyroidism, and permanent recurrent laryngeal nerve injury were found in patients who developed cervical hematoma, while rates of wound infection and transient recurrent laryngeal nerve injury were comparable. With regard to these two studies, it is important to specify that only cervical haematomas requiring surgical revision haemostasis were included, while those managed conservatively were not considered.

Our result relating to postoperative hypoparathyroidism was quite unexpected and, to our knowledge, has not been described by other authors. In our opinion, it could be explained by the fact that a less aggressive haemostasis, which caused the occurrence of cervical haematoma, at the same time favored the preservation of the vascularisation and thus of the functionality of the parathyroid glands. Concerning unilateral recurrent laryngeal nerve lesions, they were probably due to challenging haemostasis during thyroidectomy or damage during surgical revision of haemostasis, which are both situations that hinder an adequate vision of anatomical structures. Regarding this result it is important to point out that it may have been influenced by the fact that the use of intraoperative nerve monitoring, which especially in difficult cases can help the surgeon to preserve the recurrent laryngeal nerve (5), was significantly inferior in patients who developed postoperative cervical haematoma.

This study has some limitations due to the retrospective nature of our analysis. Firstly, it was not possible to analyse all risk factors reported in previous studies. The second limitation regards cervical haematomas managed conservatively. Their incidence is very likely underestimated and, moreover, it was not possible to evaluate the timing of their onset, as this information was available only for a very limited number of these cases. Finally, as regards the result concerning unilateral recurrent laryngeal nerve lesions in patients requiring surgical revision of haemostasis, it was not possible to specify whether this complication occurred as a result of the first or second surgical procedure.

## Conclusion

Postoperative cervical haematoma is an uncommon but potentially life-threatening complication of thyroidectomy.

Our study identified male sex, older age, higher BMI, unilateral lateral neck dissection, drain placement, and shorter operative times as independent risk factors for the onset of this complication. Differently, the use of energy-based devices working by means of hybrid energy was found to be protective for its development.

Based on our findings, we believe that patients with these risk factors should be closely monitored in the postoperative period, particularly during the first six hours after the operation, and excluded from outpatient surgery.

Considering the limitations of our analysis and the different results obtained by other authors, further prospective studies with large populations are needed to better investigate this topic.

## Data Availability

The raw data supporting the conclusions of this article will be made available by the corresponding author, without undue reservation.
